# Patterns of saliency and semantic features distinguish gaze of expert and novice viewers of surveillance footage

**DOI:** 10.3758/s13423-024-02454-y

**Published:** 2024-01-25

**Authors:** Yujia Peng, Joseph M. Burling, Greta K. Todorova, Catherine Neary, Frank E. Pollick, Hongjing Lu

**Affiliations:** 1 School of Psychological and Cognitive Sciences and Beijing Key Laboratory of Behavior and Mental Health, Peking University, Beijing, 100871 China; 2 Institute for Artificial Intelligence, Peking University, Beijing, China; 3 National Key Laboratory of General Artificial Intelligence, Beijing Institute for General Artificial Intelligence, Beijing, China; 4 Department of Psychology, University of California, Los Angeles, CA USA; 5 School of Psychology and Neuroscience, University of Glasgow, Glasgow, UK; 6 School of Health and Social Wellbeing, The University of the West of England, Bristol, UK; 7 Department of Statistics, University of California, Los Angeles, CA USA

**Keywords:** Social interaction, Visual expertise, Eye movements, Intention, Deep convolutional neural network (DCNN), Saliency

## Abstract

When viewing the actions of others, we not only see patterns of body movements, but we also "see" the intentions and social relations of people. Experienced forensic examiners – Closed Circuit Television (CCTV) operators – have been shown to convey superior performance in identifying and predicting hostile intentions from surveillance footage than novices. However, it remains largely unknown what visual content CCTV operators actively attend to, and whether CCTV operators develop different strategies for active information seeking from what novices do. Here, we conducted computational analysis for the gaze-centered stimuli captured by experienced CCTV operators and novices' eye movements when viewing the same surveillance footage. Low-level image features were extracted by a visual saliency model, whereas object-level semantic features were extracted by a deep convolutional neural network (DCNN), AlexNet, from gaze-centered regions. We found that the looking behavior of CCTV operators differs from novices by actively attending to visual contents with different patterns of saliency and semantic features. Expertise in selectively utilizing informative features at different levels of visual hierarchy may play an important role in facilitating the efficient detection of social relationships between agents and the prediction of harmful intentions.

**Supplementary Information:**

The online version contains supplementary material available at 10.3758/s13423-024-02454-y.

## Introduction

People are adept at perceiving goal-directed actions and inferring intentions from human actions. Although laboratory research using controlled stimuli (e.g., Heider-Simmel-type animations, 1944) has shed light on the visual processing involved in analyzing goal-oriented activities, most work has focused on how low-level visual cues, such as orientation and speed, affect social perception (e.g., Gao et al., 2009, 2010; McAleer & Pollick, 2008; Shu et al., 2018). It remains unclear how features extracted from different levels of the visual hierarchy influence social perception, and how people analyze visual contents of social stimuli in complex, real-world interactions.

We address these questions using real-life human activities recorded in videos of Closed Circuit Television (CCTV). The CCTV systems typically employ a set of cameras deployed around complex urban geography. The videos recorded by the cameras are routinely monitored by CCTV human operators in real-time to identify the presence of hostile intentions so as to allow a preemptive response that minimizes the consequences (Wallace & Diffley, 1998). Surveillance CCTV videos usually contain a large amount of visual information coupled with the high complexity of human activities (Howard et al., 2009; Hodgetts et al., 2017). Hence, CCTV operators, who have acquired extensive experience in the visual analysis of human actions in real-world scenes, likely adopt efficient strategies in information processing of social interactions.

Previous studies have compared CCTV operators to novices when performing the task of judging harmful intent from surveillance videos but have yielded mixed results. A study by Grant and Williams (2011) using twelve CCTV operators and twelve novices viewing video clips of 15 s showed no difference between groups in predicting antisocial behavior, with both performing at chance. Though performance was shown to be modulated by the degree to which observers focused on the social structure of the scenes. Another study by Troscianko et al. (2004), which used 50 professional observers and 50 novices, also found no group difference. Other studies, however, have found group differences between CCTV operators and novices in the ability to recognize and predict harmful intent. For example, behavioral data showed that CCTV operators were more sensitive than novices when making predictions about harmful intentions (Petrini et al., 2014). Consistently, the resulting brain imaging data showed reduced activity for experienced CCTV operators in the parahippocampal gyrus (Petrini et al., 2014), and in the Fusiform Face Area and the posterior Superior Temporal Sulcus (Gillard et al., 2019), which can be viewed as a sign of increased efficiency. A follow-on study from Petrini et al. (2014) using eye tracking showed that CCTV operators yielded greater in-group consistency of fixation patterns (Burling et al., 2016). Hence, studying the differences in visual processing between experienced CCTV operators and novices provides a unique window to unveil efficient strategies acquired by human experts through extensive learning.

Another special feature of intention detection from surveillance videos probes the active processing aspect in visual analysis by humans. Humans actively sample the visual input through brief fixations interspersed with gaze shifts over space and time. During a period of stable fixation, the information at the central gaze is analyzed in fine detail using foveal vision, while peripheral analysis is carried out to select the next fixation location for a gaze shift. Recent studies have shown significant differences between central and peripheral vision in the analysis of human body movements (Thurman & Lu, 2013, 2014), showing that configural cues based on the spatial arrangement of joint trajectories dominate visual processing in central vision, whereas local motion and orientation cues interact with spatial cues to influence action perception in the periphery. In addition, studies investigating surveillance videos have provided evidence that experienced CCTV operators, relative to novices, produce different goal-directed eye-movement patterns when viewing surveillance video, and show greater consistency in eye-movement tracking patterns (Howard et al., 2013; Roffo et al., 2013). Although these studies have analyzed eye-movement characteristics associated with expertise, it remains unknown what stimulus content in surveillance videos drives the active selection of gaze shifts when identifying social intentions.

Previous evidence has suggested that the understanding of human actions relies on different perspectives of visual features that unfold over time (Dima et al., 2022; Isik et al., 2020). To compare visual content attended by CCTV operators versus novices, we conducted two computational analyses that focus on different levels of visual information. The first analysis adopted a saliency model (Itti et al., 1998) to process gaze-centered regions of videos by characterizing low-level visual features including luminance, color, orientation, texture, and motion. The second computational analysis applies a deep convolutional neural network (DCNN), AlexNet (Krizhevsky et al., 2012), to extract object-level semantic features from gaze-centered regions of visual inputs (Kriegeskorte, 2015). The network architecture of DCNN is consistent with the hierarchical structure of the visual system in human brains, which enables DCNNs to cope with nonlinearity and complex visual tasks. Beyond a similar architecture, the inner representations of DCNNs have also been found to capture neural similarities in brain activities for different visual inputs (Cichy et al., 2016; Khaligh-Razavi & Kriegeskorte, 2014; Kriegeskorte et al., 2008; Yamins et al., 2013, 2014; for reviews, see Kriegeskorte, 2015; Yamins & DiCarlo. 2016). Hence, activities in later layers (e.g., fully connected layer) of DCNNs appear to capture abstract features crucial to visual knowledge and scene semantics.

Together, the Saliency model and the DCNN model provide complementary analyses for assessing how CCTV operators and novices use various features extracted from different levels of visual hierarchy. If low-level visual saliency cues have a greater impact on capturing attention and driving the inference of intentions, we would expect to find a group difference in visual saliency from the gaze-centered stimulus regions. On the other hand, if CCTV operators differ from novices primarily in the use of semantic features extracted by high-level visual processing, the DCNN may be able to capture the group differences. In addition, we examine the inter-subject correlation of visual features attended by CCTV operators and novices. If the expertise of CCTV operators leads to shared strategies that emerged from rich experience in analyzing surveillance footage, we would expect to find greater inter-subject correlation of visual features attended among CCTV operators than for novices.

## Methods

### Participants

Eleven CCTV operators (three female, aged 21–53 years, *M* = 36.3, *SD* = 10.1) and ten novices (two female, aged 28–43 years, *M* = 33.8, *SD* = 6.0) were recruited to participate in the eye-tracking experiment. The “operator” participants were all employed to monitor CCTV when the experiment was conducted and had an average of 4.5 years of working experience as a CCTV operator (*SD* = 3.0, range 0.4–12 years), and viewed CCTV an average of 10 h (range 8–12 h) per day, summing up to be roughly 10,000 h of CCTV experience on average. Only four of the 11 CCTV operators had received formal training in detecting suspicious/abnormal behavior, and that training was reported to be mainly in-house given by colleagues or on occasion from the local police. Thus, the majority of CCTV operators gained their expertise in detecting suspicious behavior through practice and viewing a large number of surveillance videos. The “novice” participants were defined as individuals with no CCTV surveillance or security experience. All participants were adults with the racial category of white. The age of the operators and novices were matched (independent t-test, *t*(19) = 0.875, *p* = 0.392). CCTV operators were recruited from CCTV control rooms and user groups. Novices were recruited from the community through advertisements.

Each participant read and signed a Consent Form that described their participation in the experiment and the use of the data collected. Informed consent was obtained from all participants. All participants were free to leave the study at any time. Ethical approval for the primary data collection phase of the study, which occurred during the period 2008–2010, was obtained from the UK Ministry of Defense Research Ethics Committee. Participants were paid for their time and travel expenses to attend the experiment at BAE Systems, Advanced Technology Centre, Bristol. All methods were performed in accordance with the Internal Review Board (IRB) guidelines and regulations and have been performed in accordance with the Declaration of Helsinki. All participant data were de-identified for the current analysis.

### Stimuli and procedure

Videos of street scenes with human actions recorded by CCTV were selected from originally over 800 h of CCTV footage obtained of urban scenes in the UK. Four paid research assistants with no prior CCTV experience screened the corpus of video material and identified CCTV clips that resulted in physical aggression (and therefore included hostile intent), which were labeled as the “Fight” clips. Control scenes were chosen for the “Confrontation”, “Playful”, and “Neutral” categories and were matched to the Fight clips in several respects: location, time of day, and the number of people in each display. A total of 36 videos were generated for the four action categories, with nine videos in each category. The same stimuli have been used in the previous study by Petrini et al. (2014). Each video lasted 16 s with a frame rate of 25 fps, yielding a total of 400 frames, with the image size of 576 × 480 pixels in a visual angle of 22.5° × 19°. Most of the people appeared in the CCTV footages were white, predominately young males. In answers to the debrief question of “what did you look for in the CCTV footage in order to help make your decision?”, none of the participants mentioned race-related information. CCTV clips obtained for all four categories were matched in terms of location, time of day, and number of people in displays. The fight clips showed 16 s of behavior prior to the onset of a violent incident. Hence, no participants in this study viewed any actual violent acts during the 16-s videos.

Participants (CCTV operators and novices) were shown the 36 CCTV footage clips with a quasi-random order, in which no clip was preceded by another clip from the same category. Their eye movements were recorded while they watched the CCTV footage clips in each trial. After each clip was shown, participants were asked to predict whether a violent event took place or not. Specifically, they rated the likelihood that the clips would end in violence, using a six-point scale that ranged from 1 (extremely unlikely) to 6 (extremely likely).

Eye movements were recorded when participants viewed these 36 videos. Eye-movement data were collected using an ASL Eye-Trace6 system with a sample rate of 50 Hz and accuracy approximately 1° across the visual field. For each participant, we extracted square image patches from each video frame with a window size of 75 × 75 pixels centered on a gaze-fixation location in each frame. The size gaze-contingent window (approximately 3°× 3°) is larger than the standard estimate of the foveola, which is roughly 0.5–1.0° in diameter (Boff & Lincoln, 1988). We also examined different window sizes of 38 × 38 (1.5° × 1.5°) and 150 × 150 (6° × 6°) and got similar results. Certain video frames had missing eye-tracking data, with an average of 1.1% (i.e., 4.6 frames among 400 frames).

### Behavioral analyses

Behavioral results of the same dataset were partially reported in (Petrini et al., 2014). A repeated-measures ANOVA was conducted on the ratings regarding the likelihood that the clips would end in violence. The analysis used the group label (novice vs. expert) as a between-subject factor, and the action category (i.e., Fight, Confrontation, Playful, and Neutral) as a within-subject factor. Detailed results are reported in the Online Supplemental Materials (OSM) S1.1.

To further examine the sensitivity and biases of intention judgments of CCTV operators and novices, a signal detection analysis was applied to the likelihood ratings of four action categories. Judging a fight clip as having a violent outcome (ratings of 4 or more) was scored as a “hit,” and judging a confrontation, playful or neutral clip as not having a violent outcome (ratings of 3 and less) was scored as a “correct rejection.” A calculation of sensitivity index d′, and criterion C, was performed for each participant, which were used in the decoding analyses below as well. Detailed results are reported in the OSM S1.2.

### Computational analyses

**Fig. 1:**
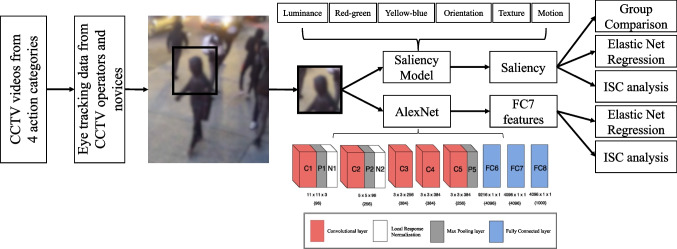
Procedures for feature extraction and comparison. A square image patch centered on coordinates of fixations was extracted from each image frame and these were fed into models as input. The saliency model extracted saliency features, which were used to derive a saliency index to compare across groups. The extracted saliency features were also entered into an elastic net regression model for decoding CCTV operators from novices. The AlexNet extracted fully-connected layer features as inputs for entry to an elastic net regression model for the decoding purpose. Inter-subject correlation (ISC) indices based on saliency features and DCNN features were compared between groups. Note, the blurred image frame was selected for demonstration and was not from the real experimental materials

Two computational models were used to extract visual features from the raw videos of surveillance footage: a saliency model and a deep convolutional neural network model. An illustration of the procedure is shown in Fig. 1. The saliency model was adopted to capture low-level image features that attract attentive gaze, and the DCNN model was adopted to capture object-level features that capture semantic information in attended visual scenes. For frames with missing eye-tracking data, blank image patches were extracted and zeros were used for computational models.

#### Saliency model and saliency index

We adopted the saliency model by Itti and colleagues (Itti et al., 1998) to analyze the influences of low-level visual cues on gaze patterns. The saliency model processes visual input in a bottom-up manner and does not capture high-level visual features associated with objects or people. The saliency model decomposes visual inputs into a set of topographic feature maps, such as motion, luminance, color, texture, and orientation (Treisman & Gelade, 1980). All feature maps feed, in a purely bottom-up manner, into a master "saliency map," which topographically codes for local conspicuity over the entire visual scene. Different spatial locations then compete for saliency within each map, such that only locations which locally stand out from their surround can persist. Specifically, the saliency model can compute scores reflecting the degree of gaze-centered regions capturing bottom-up visual attention in the video frames.

**Fig. 2:**
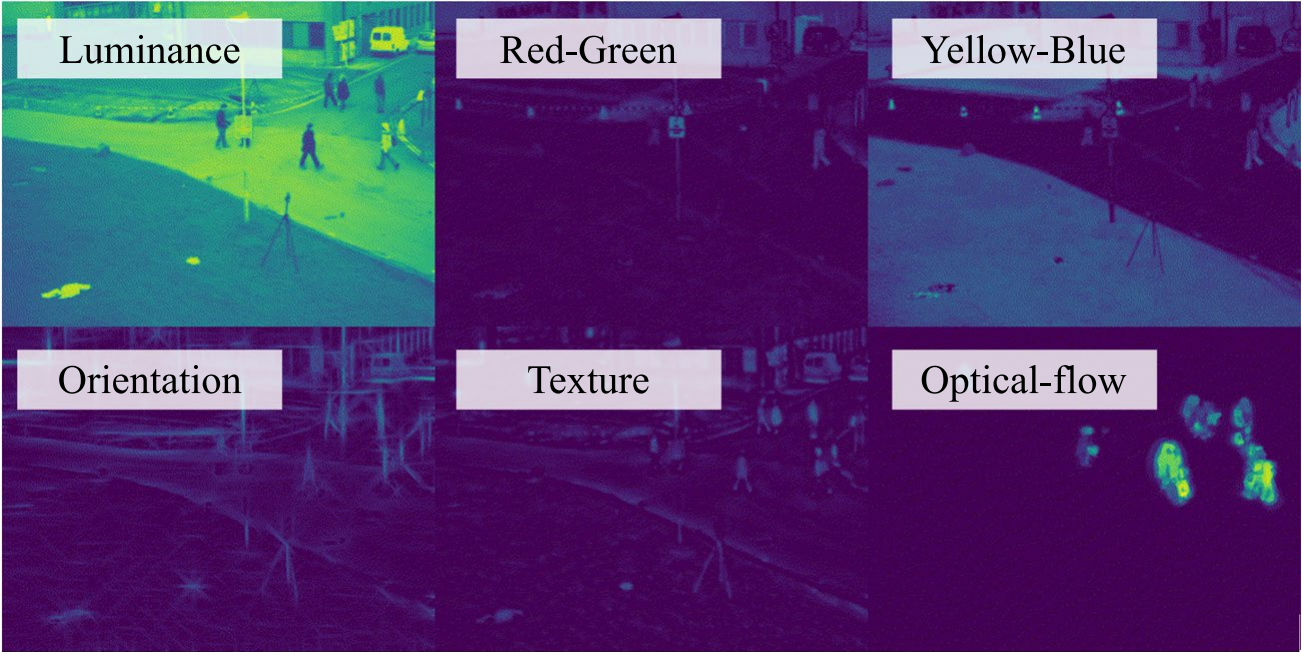
Image features were extracted from each image frame through six processing channels in the saliency model. Note, the image frame was selected for demonstration and was not from the real experimental materials

As shown in Fig. 2, image features were extracted from each image frame through six processing channels: luminance, color (red-green and yellow-blue), orientation, texture, and motion. Luminance and color maps were calculated based on the Derrington-Krauskopf-Lennie (DKL) color space (Derrington et al., 1984) using long, medium, and short cone response filters. Luminance maps were computed as the sum of long and medium cone responses. Colors were defined as the difference between long and medium for the red-green colormap and (Long + Medium) - Short for the yellow-blue colormap. The orientation map was created by applying a series of Gabor filters to the grayscale image to detect line-segment edges. The texture map was created by applying a series of Laplacian of Gaussian (LoG, or Mexican hat) filters of different spatial sizes proportional to the grayscale image size. The optical flow map was estimated using an orientation tensor (Farneback, 2000), which processes the current and the previous image frame to detect shifts in location of each pixel in temporally neighboring frames. Only vector magnitude was used to represent optical flow magnitudes (i.e., motion speed) without the consideration of motion directions.

For each image frame, a pyramid stack was created for each feature channel that included the original size of the source image patch (75 × 75 pixels), half-size, quarter-size, and eighth-size scales of the image. Gaussian blur was applied before each downsampling operation. Pyramids achieved the result of increasing the receptive field during the activation step. A center-surround activation step was implemented as a combination of Laplacian-Gaussian convolution and Gaussian blur, and was applied to each map iteratively for a total of five passes of the convolution kernels. All convolution kernels used to extract feature maps during activation were applied to the source image. A temporal buffer of the pyramid stacks was created to keep track of the previous two frames and the current frame. The buffer was used to record previous feature channel weights and final saliency maps, acting as a weighted memory for past salient regions. For each new frame, past saliency images were weighted by half of their current values.

After extracting and processing the pyramid stacks, feature maps have different value ranges. The normalization step obtains a common scale across all feature maps so that they can later be combined into a single saliency map. For normalization, each feature map within a pyramid stack was scanned to enhance the contrast between salient and non-salient regions. The sum of feature values for the salient regions was used to compute normalization factors to be applied to the set of feature maps. The normalization factors for the current source image were weighted by the normalization factors stored in the temporal buffer, and the current maps were then scaled according to the weighted normalization factors. The weighted normalization factors used for subsequent analysis were exported for each image frame to assess the relative contribution of each feature channel.

To calculate the final saliency map for an image frame, first, the feature maps were compressed into a single map by summing across pyramid levels for each channel and dividing by the size of a pyramid stack, yielding an intermediate saliency map for each feature channel. Secondly, the intermediate saliency maps per feature channel were summed to create a single saliency map for the current image frame. The saliency map for the current image frame was then combined with the weight-decayed saliency maps from previous two frames and the current frame in the temporal buffer to obtain a final saliency map. Lastly, the final map was processed with a logistic activation function that increases the contrast between salient and non-salient regions, which was also exported for analysis. Using the final saliency maps from a sequence of image frames, a *saliency index* was calculated by computing the average saliency values within a gaze-centered region in the saliency maps.

#### Saliency analysis

To examine whether CCTV operators and novices’ gaze patterns are impacted differently by low-level saliency cues, we conducted repeated-measures ANOVAs for each action category to examine the group difference between CCTV operators and novices on the saliency index and the six feature dimensions. We hypothesize that if the visual contents attended by CCTV operators in their gaze fixations differ from the visual information captured by novices in terms of salient low-level features (e.g., luminance or motion features), we would expect to observe a group difference between the two groups of participants in the saliency index obtained from gaze-centered regions derived from their eye movement patterns.

We concatenated six saliency features across frames to form a multidimensional saliency vector to train a machine learning classifier based on elastic net regularization (Tibshirani, 1996; Zou & Hastie, 2005). The classifier was trained to differentiate CCTV operators and novices based on the attended low-level saliency information. Specifically, the concatenated feature vectors were entered as predictors to the generalized linear model (GLM) to classify CCTV operators and novices. The classifier used the elastic net regularization to favor the selection of a small number of important features that help predict the class labels. The elastic net regularization has a free parameter, α, controlling the weight between a lasso (L1) and a ridge (L2) regularization. We set an alpha value of 1 to favor a smaller number of features. We also used a parameter value of 0.9 and 0.8 that yielded similar results. The model was trained in a leave-one-out manner with 21 iterations. Specifically in each iteration, 20 participants were randomly selected for training, and the remaining one participant was used for testing to let the classifier determine whether this testing participant was a CCTV operator or a novice. Classification accuracy was averaged across all 21 iterations.

To reflect the online processing with cumulative information over time, for each video, features were concatenated across a set of non-overlapping cumulative temporal windows with a step-size of 50 frames (i.e., concatenating features of frames 1–50, frames 51–100, …, and frames 351–400), yielding eight chunks of feature vectors. Using cumulative frames by concatenation takes into consideration the temporal dependency in action videos. We explored a set of temporal cumulation windows because critical events occurred at different time points for different surveillance footage.

Since the most informative signal that differentiates CCTV operators and novices may emerge at different time points, the *maximum* classification accuracy over temporal cumulation windows was used for each video as the decoding accuracy of the classifier. For example, for confrontation video No.1, the maximum classification accuracy may arise from early frames of the video with cumulating frames 1–50, while confrontation video No. 2 may reveal the maximum classification accuracy from a different temporal window of frames 51–100. If operators and novices attended to systematically different low-level features captured through the saliency model, we would expect that the classifier should show above chance-level accuracy in differentiating operators and novices. Furthermore, if attentive features were influenced by the nature of intention underlying the observed actions, the classifier accuracy may vary depending on the presence or absence of harmful intentions. Bonferroni multiple-comparison correction was applied to the statistical testing of decoding accuracy.

Furthermore, to examine whether operators or novices consistently attend to information with high saliency, inter-subject correlations were calculated for the operator group and the novice group separately. For image patches centered at gaze fixations in each frame, a saliency vector was extracted from all six feature maps. To transform features onto a common scale and remove outliers, z-score normalization was applied for each feature channel across all videos and subjects. The similarity of gaze-centered saliency between a pair of participants was computed as the correlation of concatenated saliency vectors over time for each video (i.e., each video yields a 2,400-element-long vector coming from six features of 400 frames). Higher similarity values indicate that two participants attended to regions with a similar degree of visual saliency. For each video, the inter-subject correlation (ISC) was defined as the average similarity value across all pairs of participants within the operator group and within the novice group.

#### Deep convolutional neural network model and analysis

In the DCNN analysis, due to the high similarity of objects involved in consecutive frames, one frame out of every ten frames was sampled as inputs into models. Thus, the original 400 frames of surveillance footage were downsampled to 40 frames to reduce computational demands. To investigate the group difference on a semantic level, we adopted a pre-trained DCNN, AlexNet, to extract object-level features. AlexNet contains five convolutional layers and three fully connected layers. For each image patch centered at the gaze fixation, we extracted the activations from the penultimate layer, fully connected layer 7 (FC7, containing 1*4096 units), a layer just before the decision layer for object categorization in AlexNet. For each video (36 videos in total), features of image patches centered on fixations were extracted from the penultimate layer (i.e., FC7) of AlexNet. Each gaze-centered image patch yields a feature vector in a size of 1 by 4096. Similar to the analysis approach used for the saliency model, to reflect the online processing with cumulative information over time, for each video, features were concatenated across a set of cumulative frame windows with a step-size of 5 frames (i.e., concatenating features of frames 1–5, frames 6–10, …, and frames 36–40). Because the DCNN features were downsampled by a factor of 10, current windows with five frames match what was used for the saliency model. The classifier with elastic net regularization was applied to the DCNN features to differentiate visual information attended by CCTV operators and novices. Training and testing procedures were the same as the classifier with saliency features. If operators and novices attended to systematically different object-level features captured through the DCNN model, we would expect the classifier to show the above chance-level accuracy in differentiating operators and novices.

Similar to the saliency analysis, to examine whether operators or novices consistently attend to object-level information extracted by DCNN, inter-subject correlations were calculated for the operator group and the novice group separately. First, FC7 features of all gaze-centered image regions were concatenated across time by video. ISC was calculated as the correlation coefficients of concatenated feature vectors between pairs of operators, or between pairs of novice participants. This procedure was repeated for each of the 36 videos, respectively.

#### Analysis based on the combination of Saliency and DCNN features

To examine joint influences of low- and high-level information that supports intention inference and differentiates CCTV operators from novices, we examined the decoding performance of differentiating two groups by each feature type separately, as well as jointly by concatenating both types of features. The decoding was conducted frame-by-frame, and the maximum accuracy was taken from each 4-s time-chunk.

Furthermore, to examine how visual features associate with behavioral performance, we performed an action-category decoding analysis with both types of features. Specifically, the elastic net performs decoding of fighting versus non-fighting videos (i.e., confrontation, playing, and neutral actions) for each subject. The decoding analysis was conducted frame-by-frame, and the maximum accuracy was taken from each 4-s time-chunk. Pairwise Pearson correlations were conducted between behavioral performance (i.e., sensitivity and bias) and decoding accuracies of two types of features.

Finally, to examine how the two types of features differ between operators and novices, we conducted an ISC analysis between CCTV operators and novices. For each video, an average saliency/DCNN feature vector was calculated for the operator group and the novice group respectively. Correlations between the individual-subject feature vector and the averaged group feature were calculated (i.e., individual operators/novices vs. averaged operator/novice group features), yielding degrees of similarity between CCTV operators and novices.

#### Looking content analysis based on AlexNet outputs

We further explored AlexNet output layers of 1,000 object categories to examine the characteristics of looking contents in gaze-centered regions. Please see OSM S4 for the detailed analyses methods and results.

## Results

### Saliency analyses

#### Saliency indices of CCTV operators and novices

**Fig. 3:**
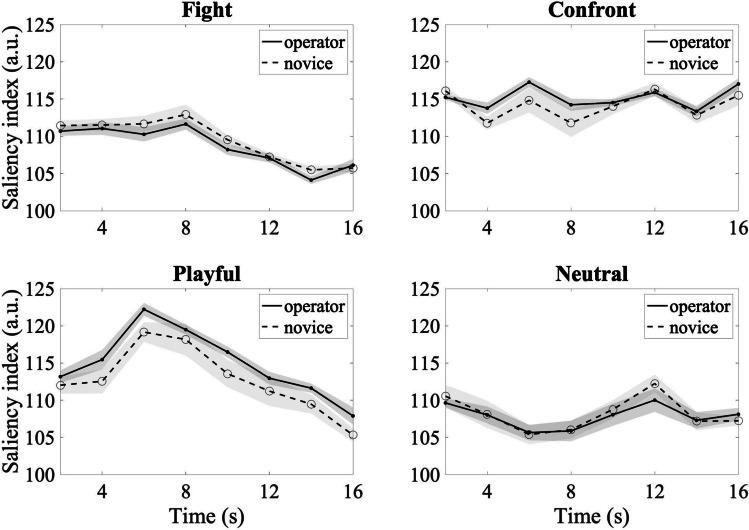
Saliency indices of the CCTV operator and novice groups over time for four types of actions. Each data point corresponds to the averaged saliency index every 2 s. Shaded areas indicate standard error

Figure 3 depicts the saliency index for image patches in the gaze fixation areas as a function of video time for CCTV operators and novices. Mixed ANOVA models with participant group as a between-subjects factor and time as a within-subjects factor were conducted on the saliency index for each of the four action categories. For all four types of actions, we found significant main effects of time (*p*s *<* .001). This result suggests that visual saliency changes dynamically across video frames. However, no action type showed a main effect of participant group, revealing that image patches attended by CCTV operators and novices do not show significant differences in terms of total visual saliency scores. The two-way interaction effect between time and participant groups was not significant for any of the action types (*ps* > 0.05). We also reran the analysis by removing missing frames and results yielded a similar trend (see OSM S2.1).

#### Saliency feature decoding of CCTV operators and novices

**Fig. 4:**
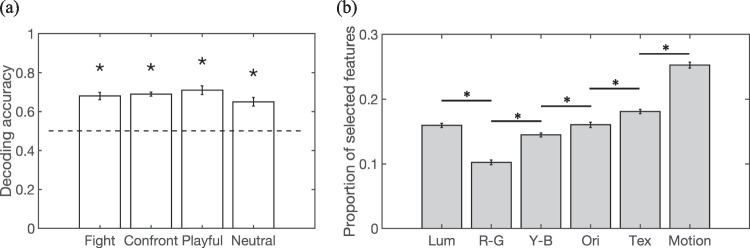
(**a**) Decoding accuracy based on saliency features for discriminating CCTV operators from novices. Error bars indicate SDs of accuracy from leave-one-out iterations. Asterisks indicate significantly greater than the chance level tested against a Bonferroni-corrected alpha level. (**b**) Proportions of saliency features selected in the elastic net regression decoding analysis. From left to right, the six feature dimensions represent luminance, red-green color, yellow-blue color, orientation, texture, and optical-flow motion information. Error bars indicate SDs of decoding accuracies among videos in each action category. Asterisks indicate significant differences between feature dimensions

We next conducted a multivariate classification analysis to investigate whether CCTV operators and novices could be decoded based on patterns of saliency features. As shown in Fig. 4a, all four actions reached decoding accuracy that was significantly greater than the chance level of 0.5. One-sample t-tests were carried out and each tested against a Bonferroni-adjusted alpha level of 0.0125 (0.05/4 for four action categories, same below, Fight: *M* = 0.68; Confrontation: *M* = 0.69; Playful: *M* = 0.71; Neutral: *M* = 0.65, *p*s < .01). To ensure that the unequal sample size did not affect the reported results, we also ran analyses by removing one data point from the CCTV operator group. Results were similar to before, where all four actions reached a decoding accuracy that was significantly greater than the chance level tested against a Bonferroni-adjusted alpha level (see OSM S2.2 for detailed reports). By examining the contribution of six saliency features, we found that optical-flow motion information was the most frequently selected feature to differentiate operators from novices (see Fig. 4b and OSM S2.3).

To examine whether the effect was driven by noise, we conducted a permutation test where the feature dimension of saliency channels was shuffled, and decoding was conducted based on shuffled data. A total of 100 iterations were conducted and the distribution of permuted decoding accuracy results was compared to the real effect. One-sample t-tests showed that the averaged decoding accuracy based on real saliency features were significantly greater than the performance of permuted results for all action categories (*p*s < .001).

#### Inter-subject correlation (ISC) of saliency index

**Fig. 5:**
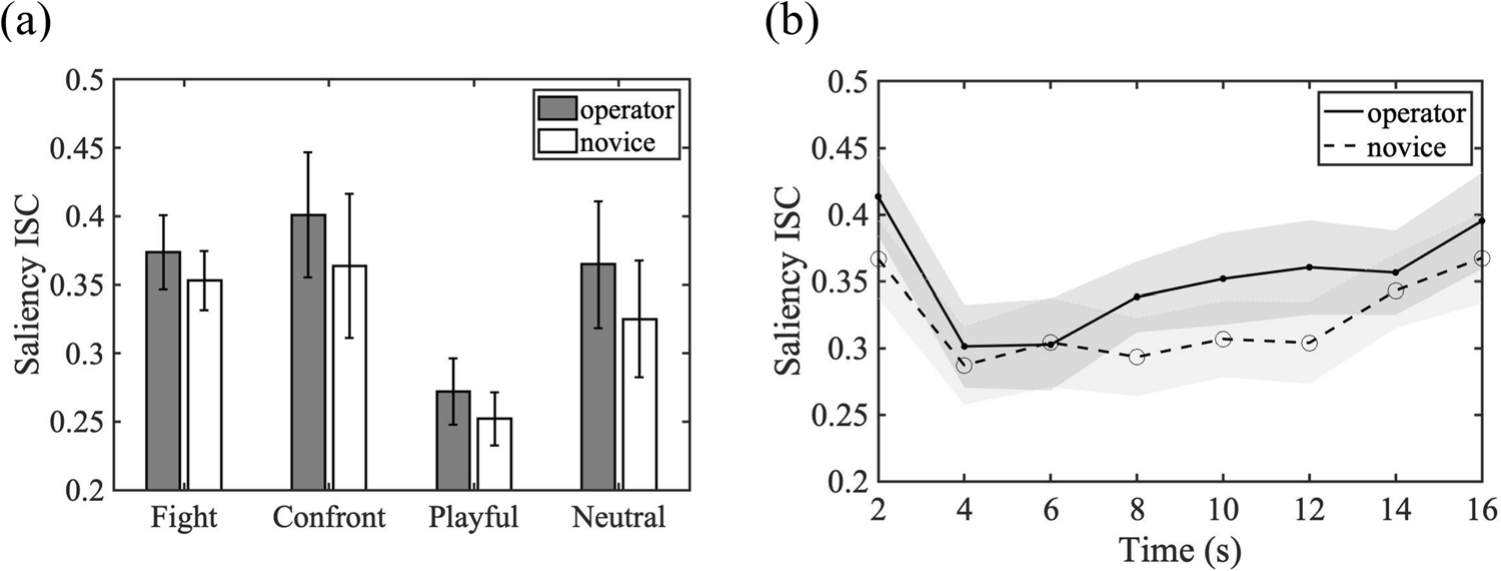
Saliency inter-subject correlation (ISC) results. (**a**) Saliency ISC of each action category within the CCTV operator group and within the novice group, calculated by averaging pairwise correlations of saliency features concatenated over time for each video. Error bars indicate SEs across videos in each action category. (**b**) Saliency ISC integrated within every 2-s time window for CCTV operators and novices. Shaded areas indicate SEs

Saliency ISC of each action category within the groups are shown in Fig. 5a. A repeated-measure ANOVA was conducted with groups and action categories as within- and between-subject factors. The ANOVA showed a significant main effect of the participant group, *F*(1,32) = 9.38, *p* = 0.004, *η*_*p*_^*2*^ = .227, observed power = .844, resulting from greater inter-subject correlation among experienced operators (*M±SD* = 0.35±0.13) than among the novice group (*M±SD* = 0.32±0.12) . Group comparisons of ISC of each action category are reported in OSM S2.4. We also conducted Bayesian analysis on the inter-subject correlations, yielding a Bayes factor of 15.14, suggesting that the hypothesis of greater inter-subject correlation of using saliency features among operators than novices was 15 times more likely than the alternative hypothesis where two groups yielded equal ISCs on saliency features. Neither the main effect of action categories, nor the two-way interaction between groups and action categories was significant (*p*s > 0.05).

Additionally, to examine how the group difference on the consistency in the extracted saliency features emerges over time, we examined the dynamic change of ISC for every two seconds in time, yielding eight time-chunks. Saliency ISC of CCTV operators were compared to novices at each time point. As shown in Fig. 5b, none of the group differences survived Bonferroni correction.

### DCNN feature analysis

#### DCNN decoding of CCTV operators and novices

**Fig. 6:**
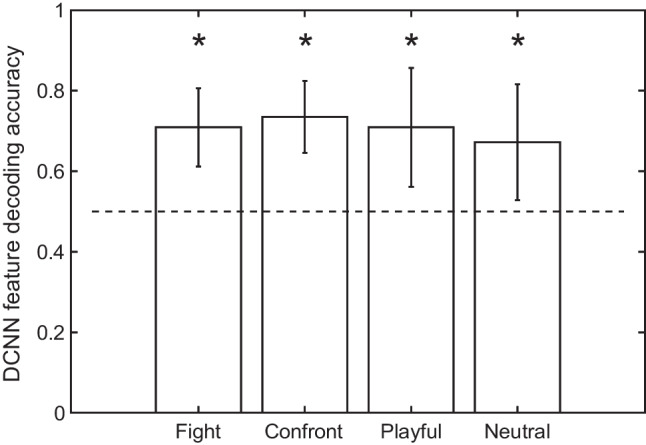
Decoding accuracy based on fully-connected layer DCNN features on discriminating CCTV operators from novices. Error bars indicate SDs of accuracy among all videos in one action category. Asterisks indicate significantly greater than chance level tested against a Bonferroni corrected alpha level

Features extracted from DCNN were used to train a classifier to recognize visual information attended by operators or by novices. As shown in Fig. 6, all actions reached a classification accuracy that was significantly above the chance level after Bonferroni correction (Fight: *M* = 0.71; Confrontation: *M* = 0.74; Playful: *M* = 0.71; Neutral: *M* = 0.67, *p*s < .01), suggesting that the DCNN features for gaze-centered regions were able to classify CCTV operators from novices. Permutation tests further showed that the averaged decoding accuracy based on real DCNN features were significantly greater than the performance of permuted results for all action categories (*p*s<.001). We also reran analyses by randomly removing one data point from the CCTV operator group to equate the sample size of the two groups, and all four actions yielded decoding accuracy that was significantly greater than the chance level tested against a Bonferroni-adjusted alpha level (see OSM S3.1 for a detailed report). We also reran the analysis by removing two subjects with excessive missing frames and results yielded a similar trend (see OSM S3.2).

#### Inter-subject correlation of DCNN features

**Fig. 7:**
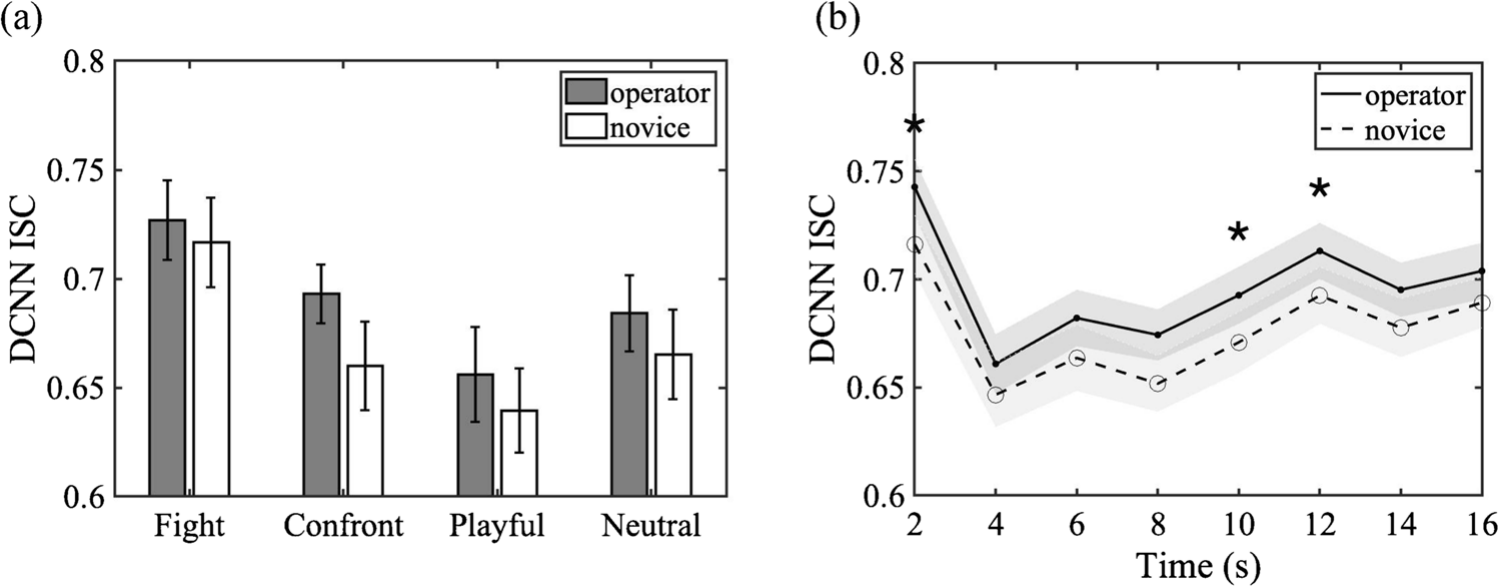
DCNN ISC results. (**a**) DCNN ISC of each action category as calculated by averaging pairwise correlations of DCNN FC7 features concatenated over time within the CCTV operator group or within the novice group. Error bars indicate SEs across videos in each action category. (**b**) Group differences in DCNN ISC over time. Shaded areas indicate SEs. Asterisks indicate significant group differences tested against a Bonferroni corrected alpha level

As shown in Fig. 7a, using DCNN features, ISCs averaged across nine videos in each action category were compared between CCTV operators and novices. A repeated-measure ANOVA showed a significant main effect of the participant group, *F*(1,32) = 26.26, *p* < .001, *η*_*p*_^2^ = .451, observed power = .999, resulting from higher inter-subject correlation among experienced operators (*M ± SD* = 0.69 ± 0.062) than among the novice group (*M ± SD* = 0.67 *±* 0.067). Bayesian analysis yielded a Bayes factor of 2836.03, suggesting that the hypothesis of greater ISC among operators than novices was 2,836 times more likely than the alternative hypothesis of no group differences. The main effect of action categories was not significant, *F*(1,3) = 2.31, *p* = .095, *η*_*p*_^2^ = .53. The two-way interaction between groups and action categories was not significant, *F*(3,32) = 1.56, *p* = 0.219, *η*_*p*_^2^ = .37.

To examine how the group difference on the consistency in the extracted DCNN features emerges over time, we further examined the dynamic change of ISC for every 2 s in time, yielding eight time-chunks. As shown in Fig. 7b, CCTV operators and novices showed significant group differences both at the very beginning of videos (*p* < .001, tested against a Bonferroni-adjusted alpha level = 0.00625 given eight time-chunks) and during the latter half of video displays (8–10 s *p* = .002, and 10–12 s *p* = .004). This indicates that CCTV operators showed more consistency in DCNN features than novices even at the onset of videos. This result suggests that operators may share some potential strategies to capture certain high-level semantic information about surveillance footages at the beginning and during a period later in the videos, which is critical for the recognition and prediction of intentions and potentially harmful behaviors.

### Analysis based on the combination of Saliency and DCNN features

#### Feature contributions to differentiating CCTV operators from novices

**Fig. 8:**
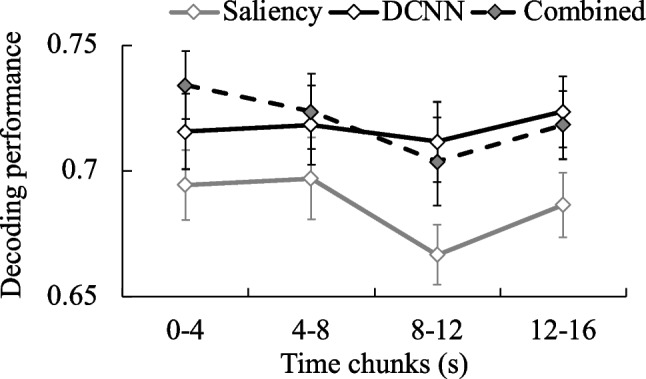
Decoding performance for classifying CCTV operators and novices based on saliency, DCNN, and combined features in gaze-centered stimulus regions over time

A repeated-measures ANOVA was performed to examine the decoding accuracy in classifying group membership of participants associated with two types of features. Action categories were entered as the between-subject variable. Types of features (i.e., saliency, DCNN, or concatenated feature) and time chunks were entered as within-subject variables. Results showed a significant main effect of feature types, *F*(2,31) = 4.48, *p* = .020, *η*_*p*_^2^ = .18. None of the other main effects, two-way, or three-way interactions were significant. Post hoc pairwise comparisons showed that the DCNN feature-only (*M* ± *SD* = .717 ± 0.009, *p* = .010) and concatenated features (*M* ± *SD* = .720 ± 0.008, *p* = 0.005) yielded significantly greater decoding accuracies than the saliency-only feature (*M* ± *SD* =.686 ± 0.007). Concatenated features did not show a significant difference compared to DCNN-only features (*p* = .695) in terms of decoding accuracy in classifying CCTV operators and novices. The results indicate that DCNN features capture high-level information that may be used differently between CCTV operators and novices, in addition to the different use of low-level visual features between the two groups. Interestingly, as shown in Fig. 8, decoding accuracy in classifying group membership showed greater differences between DCNN and saliency features over time, especially in the latter half of video observations (8–12 s, *t*(35) = 2.096, *p* = .043, and 12–16 s, *t*(35) = 2.057, *p* = .047). Together, the decoding accuracy of the combined features consistently revealed that CCTV operators may use both saliency and DCNN features differently from novices at the beginning of the videos (as combined features showed greater decoding accuracy than either saliency or DCNN features in the decoding of groups), but the group differences primarily emerged to object-level DCNN features during the second half of the videos as events unfold over time (as DCNN features started to show significantly greater decoding accuracy in the latter half than the saliency features).

#### Inter-group correlations of saliency and DCNN features

**Fig. 9:**
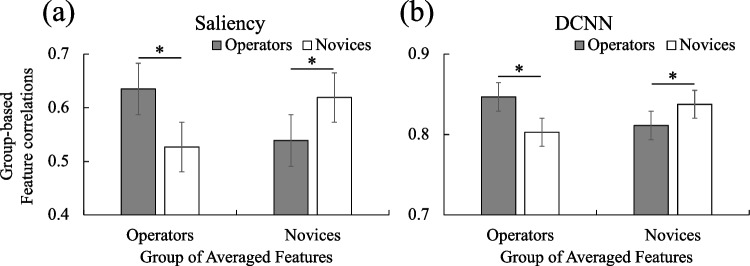
Inter-group correlations for (**a**) Saliency features, and (**b**) DCNN features. Averaged features were calculated for each subject group, for each video respectively. Pearson correlations were conducted between individuals in each group and the averaged group features. Error bars indicate SDs of correlation coefficients among all the videos

Correlations between two groups were entered into repeated-measure ANOVA. Not surprisingly, for saliency features, results showed a significant two-way interaction between individual feature groups and averaged feature groups, *F*(1,35) = 425.78, *p* < 0.001, *η*_*p*_^2^ = .924, observed power = 1.0, showing that individual features showed significantly greater correlations to the averaged features of the same group assignment (Fig. 9a). For DCNN features, a similar two-way interaction effect was found, *F*(1,35) = 720.78, *p* < 0.001, *η*_*p*_^2^ = .954, observed power = 1.0 (Fig. 9b). Both CCTV operators and novices got a fair level of correlations to the averaged features from the other group, but significantly greater correlations within groups. The patterns of results suggest that CCTV operators and novices demonstrate shared and unique features components on both low-level saliency cues and high-level DCNN features.

## Discussion

The current study adopted a saliency model and a DCNN model to examine the impact of low- and high-level visual information attended in gaze patterns of experienced CCTV operators and novices when viewing surveillance footages. For the low-level visual cues extracted by the saliency model, we did not find group-level differences in saliency indices, but classifiers based on patterns of saliency features of gaze-centered regions distinguished CCTV operators from the novices. We also found greater consistency of using saliency information among CCTV operators, suggesting that CCTV operators employed shared strategies to focus on certain patterns of visually salient cues (e.g., certain motion patterns) that likely facilitated intention inference and prediction. For the object-level features extracted by the DCNN model, we were also able to distinguish the gaze patterns of the CCTV operators from the novices, which reflect semantic representations of entities in visual scenes. Additionally, CCTV operators showed a higher inter-subject correlation in using similar DCNN features than novices, suggesting more similar information-seeking eye-movement patterns among operators when predicting potentially harmful interaction outcomes.

Here, we reliably decoded groups based on both patterns of low-level saliency features and patterns of object-relevant semantic features extracted by DCNN. These results may suggest that extensive experience of monitoring surveillance footage induces strategies in different patterns of gaze-centered saliency and semantic features toward goal-directed actions. For example, Howard et al. (2010) found that individuals with more experience watching football matches made eye movements to goal-relevant areas of the scene earlier than non-experts. In a meta-analysis by Gegenfurtner et al. (2011), effects of expertise were robustly associated with an increased frequency of fixations on goal-relevant information and reduced latencies for first fixations on these areas. From the decoding of saliency features, we found that the most frequently used feature that distinguishes CCTV operators and novices was motion cues, which may result from efficient processing of human actions in experts. The enhanced attention to goal-relevant information (and consequently, reduced attention to irrelevant information) may underly the effect of expertise in a variety of visual tasks (Haider & Frensch, 1996).

The higher inter-subject correlation among operators in the extracted saliency features and DCNN features based on looking behavior is consistent with previous findings about expertise in processing surveillance footages. For example, Howard et al. (2013) found that when monitoring a single scene to detect potentially suspicious events, trained CCTV operators showed greater consistency in fixation location by "knowing what to look for" compared to novices. Using the same dataset as the current study, Roffo et al. (2013) found that expert operators are more likely to focus on a small number of interesting regions, sampling them with high frequency. A neuroimaging study also provided converging evidence by demonstrating that CCTV operators showed increased synchronization of neural responses in certain regions of the brain than do novices (Petrini et al., 2014).

By contrasting the two types of model features, we found evidence that the high-level DCNN feature may contribute more to differentiating looking behaviors of CCTV operators from novices. As shown in OSM S4, the AlexNet output analysis, while CCTV operators showed greater probabilities of looking at facial and clothing areas, novices may be distracted by texture and color information in the video clips (OSM Fig. S5). Thus, the high-level information may enable CCTV operators to have a better chance of visually locating instigators in videos that end up with violent intentions, whereas novices may be distracted by objects with high saliency, such as streetlights or moving traffic. Furthermore, by examining the change of decoding accuracy over time and relationship between decoding accuracy and behavioral performance, we showed that the two types of features may dominate visual observation at different temporal stages. While saliency cues may contribute more at early stages, DCNN features may demonstrate stronger dominance during the latter half of video observation, suggesting that intention inference may start with low-level visual cues and gradually move on to semantic-level visual processing.

A few limitations should be addressed in future studies. The fully connected layer of DCNN takes increasingly complex visual feature patterns extracted by a sequence of convolutional layers and develops invariant representations of objects that resemble the inferior temporal (IT) cortex (e.g., Cichy et al., 2016; D. L. Yamins et al., 2014). However, even though the AlexNet model was pre-trained to recognize 1,000 object categories, it does not contain all the entities often encountered in surveillance footage, and may restrain the formation of efficient representations of agents and objects. Future studies with DCNNs that are more specialized in video understanding and scene analysis may further advance the probe of high-level semantic information contributing the expert’s recognition of intentions in social interactions. For example, the two-stream DCNN (Simonyan & Zisserman, 2014) inspired by the two-stream processing of biological motion perception in the brain provided a qualitative account of some behavioral results observed in human biological motion perception (Peng et al., 2021) and may be used in future investigations.

Together, the current study combines eye movement data with computational analysis to reveal the impact of intensive training with surveillance footage on the visual processing of human interactions from a unique perspective. The results from the two computational analyses indicate that CCTV experience facilitates the recognition of intention from natural videos via actively processing low-level visual saliency and object-level semantic information. Novices may be momentarily distracted by unimportant visual cues that do not necessarily inform the upcoming social outcomes. In contrast, CCTV operators may consistently and strategically direct selective attention toward visual regions revealing goal-relevant semantics, such as a person walking toward a group of people who may end up joining the fight. Indeed, part of CCTV training includes developing awareness for a whole scene to acquire evidence about all relevant people and objects (Walker et al. 2021). The current results not only shed light on how extensive experience shapes up visual processing of complex stimuli in biological systems, but also illustrate the promise of using computational models to analyze visual information attended by different groups of participants. Furthermore, our findings imply that computer vision algorithms that incorporate both visual pattern recognition in images and semantic encoding of the inter-person relationship at the abstract level may advance the ability of AI in inferring social intentions and making predictions on harmful outcomes.

### Supplementary information

ESM 1 (DOCX 4389 kb)

## Declarations

### Data statement

Due to the sensitive nature of the CCTV footage used in this study, raw CCTV footage data and the used stimuli will remain confidential and cannot be shared. The dataset with visual features extracted using deep learning models and saliency indices for the gaze-centered regions used and analysed during the current study is available via the Open Science Framework at https://osf.io/64am8/?view_only=b4cd298e4cc746c5a5b5f8edd77ac9de. None of the experiments were preregistered.
