# Supplementary material for: Patterns of saliency and semantic features distinguish gaze of expert and novice viewers of surveillance footage
Source: Psychon Bull Rev. 2024 Jan 25;31(4):1745–58. doi: 10.3758/s13423-024-02454-y (PMC11358171; doi:10.3758/s13423-024-02454-y)
Supplement: Supplementary file 1 — (DOCX 4389 kb) [file 13423_2024_2454_MOESM1_ESM.docx]

**Supplemental Materials for “Patterns of Saliency and Semantic Features Distinguish Gaze of Expert and Novice Viewers of Surveillance Footage”**

Yujia Peng, Joseph M. Burling, Greta K. Todorova, Catherine Neary, Frank E. Pollick, Hongjing Lu

Supplemental Results

# S1. Behavioral analyses

## S1.1 Behavioral comparisons across CCTV operators and novices

Results showed a main effect of participant group, *F*(1, 19) = 7.11, *p* = .015, *η_p_^2^*=.272, observed power = .716, suggesting that the CCTV operators rated the clips as being likely to end in violence more often than the novices did. The main effect of action categories was also significant (*p* < .001), whereas the two-way interaction between group and action categories was not significant (*p* = .346). As shown in Figure S1, post-hoc group comparisons for each action category showed that, in comparison with novices, CCTV operators rated that fight clips (*t*(19) = 2.543, *p* = 0.020) and play clips (*t*(19) = 2.420, *p* = 0.026) were more likely to end in violence, and no group differences for confront (*p* = 0.751) or neutral clips (*p* = 0.053) were found, though none of these pairwise comparisons survived FDR multiple-comparison corrections.

Furthermore, we compared the sensitivity index d′ and criterion C between CCTV operators and novices using independent-sample t-tests. Neither the sensitivity index d′ (*M_operator_* = 1.07±0.46, *M_novice_* = 0.78±0.44, *t*(19) = 1.466, *p* = 0.159) or the bias C (*M_operator_* = 0.04±0.38, *M_novice_* = -0.26±0.34, *t*(19) = 1.869, p = 0.077) yielded significant group differences.

***Figure S1****: Behavioral results of likelihood ratings of a violent incident occurring after the clip ended show significant group differences for fight and playful action categories, though these did not survive FDR multiple-comparison corrections.*

## S1.2 Associations between model features and behavior performance

To examine how visual features associate with behavioral performance, we performed an action-category decoding analysis with both types of features. Specifically, the elastic net performs decoding of fighting vs. non-fighting videos (i.e., confrontation, playing, and neutral actions) for each subject. The decoding analysis was conducted frame-by-frame, and the maximum accuracy was taken from each 4s time chunk. Pairwise Pearson correlations were conducted between behavioral performance (i.e., sensitivity and bias) and decoding accuracies of two types of features.

As shown in Figure S2, results showed a significant positive correlation between d’ and early saliency feature decoding accuracy (the first 4s, *r* = .453, *p* = .039), as well as a positive correlation with middle-late DCNN feature decoding accuracy (the 3^rd^ time chunk of 4s, *r* = .549, *p* = .010). The exploratory findings may indicate that the sensitivity of discriminating violent vs. non-violent intentions may be more strongly supported by saliency cues at the beginning stage, while semantic-level features contribute to the inference of violent intention during the latter half of video observations.

***Figure S2****: Pearson correlations between decoding accuracies of two types of features and behavioral sensitivity (d’). Asterisks indicate significantly greater than chance level (* p<.05, ** p<.01). Results showed a positive correlation between sensitivity and the maximum decoding accuracy of saliency features during the first 4s of video observations. Also, a significant positive correlation was observed between sensitivity and the maximum decoding accuracy of DCNN features during the 3^rd^ time chunk of 4s.*

# S2. Additional analysis on Saliency indices of CCTV operators and novices

## S2.1 Group comparisons after controlling for missing frames

To examine the robustness of the results and to make sure the effects were not driven by a difference in the proportion of missing frames between operators and novices, we reran the analysis after removing missing frames. Similar results to that obtained with the complete dataset were found for all four action categories. For all four types of actions, we found significant main effects of time (*p*s < .001), but neither the main effect of participant group nor the two-way interaction effect between time and participant groups were significant (*p*s > 0.05).

## S2.2 Saliency feature decoding of CCTV operators and novices after downsampling

Because there were 11 CCTV operators and 10 novices, to ensure the unequal sample size did not affect the reported results, we also reran the decoding analyses of saliency features by removing one data point from the CCTV operator group, to make the sample size equal between CCTV operators and novices. The process was repeated 11 times, with each one of the 11 operators being removed once. The decoding accuracy was analyzed across all the downsampling iterations. All the other aspects of the decoding pipeline remain the same as before.

The analysis with downsampling yielded similar decoding results as before, where all four actions reached decoding accuracy that was significantly greater than the chance level tested against a Bonferroni-adjusted alpha level of 0.0125 (0.05/4 for four action categories, Fight: *M*=0.67 (*SD*=0.02), p < 0.001; Confrontation: *M*=0.65 (*SD*=0.03), p < 0.001; Playful: *M*=0.70 (*SD*=0.04), p < 0.001; Neutral: *M*=0.65 (*SD*=0.03), *p* < 0.001).


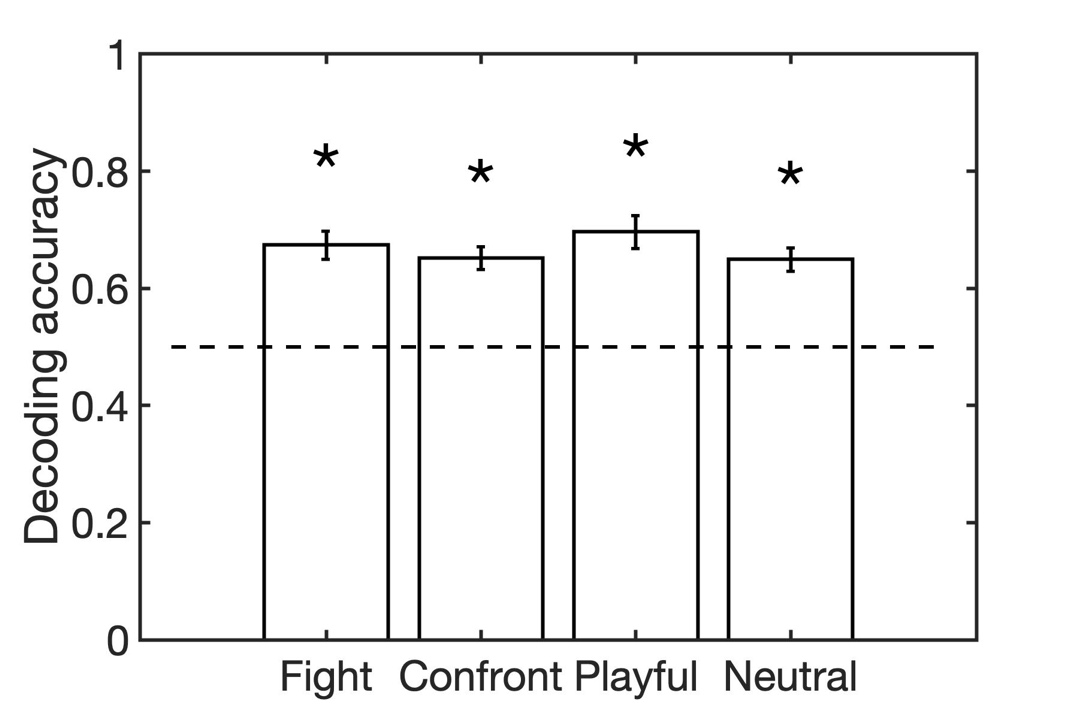


***Figure S3****: Decoding accuracy based on saliency features for discriminating CCTV operators from novices. Error bars indicate SDs of accuracy from leave-one-out iterations. Asterisks indicate significantly greater than the chance level tested against a Bonferroni corrected alpha level.*

## S2.3 Saliency feature decoding of CCTV operators and novices

To examine the contribution of six saliency features in the decoding algorithm, we calculated the proportion of features being selected by the elastic net regression model. Optical-flow motion information was the most frequently selected feature to differentiate operators from novices (*M* = 0.25, SD = 0.005), followed by texture (*M* = 0.18, SD= 0.003), orientation (*M* = 0.16, SD = 0.004), luminance (*M* = 0.16, SD = 0.003), and yellow-blue color (*M* = 0.15, SD = 0.003). This suggests that optical-flow motion information contributed the most to the classification of the two participant groups.

## S2.4. Inter-subject correlation (ISC) of saliency index

To further understand the main effect of group, the ISC of each action category was then compared between operators and novices. Playful actions showed a significant simple main effect of group difference, *F*(1,32) = 4.30, *p* = 0.046 , *η_p_^2^* = .118. None of the other three action categories reached a significant group difference on the ISC of saliency index (Fight, *F*(1,32) = 1.15, *p* = .292, *η_p_*^2^ = .035; Confrontation, *F*(1,32) = 3.77, *p* = .061, *η_p_*^2^ = .105; Neutral, *F*(1,32) = 1.08, *p* = .307, *η_p_*^2^ = .033).

To examine the robustness of the results, we reran the analysis with videos clips with more than 10% of the data points missing removed from analysis. Similar results to that obtained with the complete dataset were found after removing missing data. The main effect of the participant group was significant (*p* = 0.011), showing greater saliency ISC among experts than novices. The main effect of action categories (*p* = 0.810) and two-way interaction between groups and action categories was not significant (*p* = 0.328).

# S3. Additional analysis on Saliency indices of CCTV operators and novices

### **S3.1 DCNN decoding of CCTV operators and novices after downsampling**

Similar to the downsampling analysis of saliency features as in section 2.3, we also reran analyses of DCNN feature decoding by randomly removing one data point from the CCTV operator group to make equal sample sizes of two groups, and all four actions yielded decoding accuracy that was significantly greater than the chance level tested against a Bonferroni-adjusted alpha level after controlling for downsampling iterations and action categories (0.05/44 for eleven downsampling iterations and four action categories, Fight: *M*=0.65 (*SD*=0.05), *p* < 0.001; Confrontation: *M*=0.63 (*SD*=0.04), *p* < 0.001; Playful: *M*=0.62 (*SD*=0.03), *p* < 0.001; Neutral: *M*=0.57 (*SD*=0.07), *p* < 0.001).


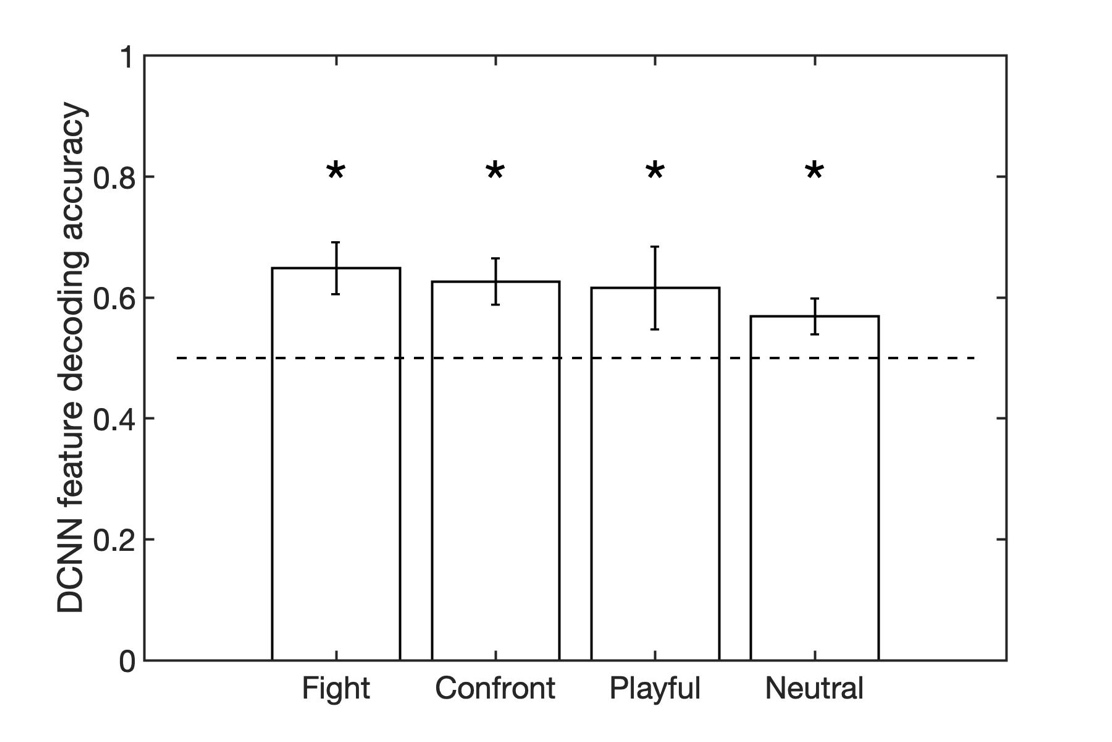


***Figure S4****: Decoding accuracy based on fully-connected layer DCNN features on discriminating CCTV operators from novices. Error bars indicate SDs of accuracy from leave-one-out iterations. Asterisks indicate significantly greater than the chance level tested against a Bonferroni corrected alpha level.*

### **S3.2 Removing subjects with excessive missing data**

We also examined the robustness of the decoding results by removing two subjects whose gaze sequences yielded excessive missing data. The decoding results with 19 subjects showed a similar trend of the results as the findings reported in the main text. However, only fighting actions reached a significant classification accuracy (*M* = 0.70, *SD* = 0.10, *p* < 0.001), surviving a Bonferroni-adjusted alpha level, whereas other action categories did not survive the multiple comparison correction (Confrontation: *M* = 0.67, *SD* = 0.15, *p* = 0.009; Playful: *M* = 0.60, *SD* = 0.10, *p* = 0.024; Neutral: *M* = 0.65, *SD* = 0.14, *p* = 0.013).

# S4. Looking content analysis based on AlexNet outputs

We further explored AlexNet output layers of 1000 object categories to examine the characteristics of looking contents in gaze-centered regions. Note that object categories in AlexNet output layer do not include a category explicitly termed as “person”, although images in some object categories are more likely to include people (such as category of “Windsor tie”, “ski”, “baseball player”, etc.). Hence our analysis examined the distribution of object categories that differentiate the most for visual contents in gaze-centered stimuli between CCTV operators and novices.

For gaze-centered stimulus sequences from an individual participant for each video clip, the maximum probability for each of the 1000 categories of AlexNet outputs were calculated over 40 frames. As a result, the distribution of object categories recognized by AlexNet for gaze-centered stimuli was derived for each participant and each video. We then computed the average category probability across all 36 videos and across participants in each group (CCTV operators vs. novices). Next, we calculated the differences in category probability distributions between the CCTV operator group and the novice group. Based on the group contrasts of object probabilities, we rank-ordered the 1000 output categories. We then visualized the top object categories where CCTV operators showed greater probabilities than novices, and the top categories where novices showed greater probabilities than CCTV operators (Figure S3). The representative images for each selected category are from the website <https://salient-imagenet.cs.umd.edu/explore.html>.

Results revealed a relatively clear distinction between the two groups: the top object categories showing higher probabilities for stimuli attended by novices than CCTV operators were visual stimuli associated with texture, color, and manmade objects, such as shower curtains, velvet, and bolo tie. In contrast, the top categories more often shown in gaze-centered stimuli from CCTV operators than from novices were faces, bodies, cloths, and animals. These results indicate that CCTV operators showed greater probabilities of looking at facial and clothing-related visual cues that may support a more efficient detection of instigators, whereas novices may be more likely distracted by low-level visual cues with high saliency of texture and color information in the surveillance footages.


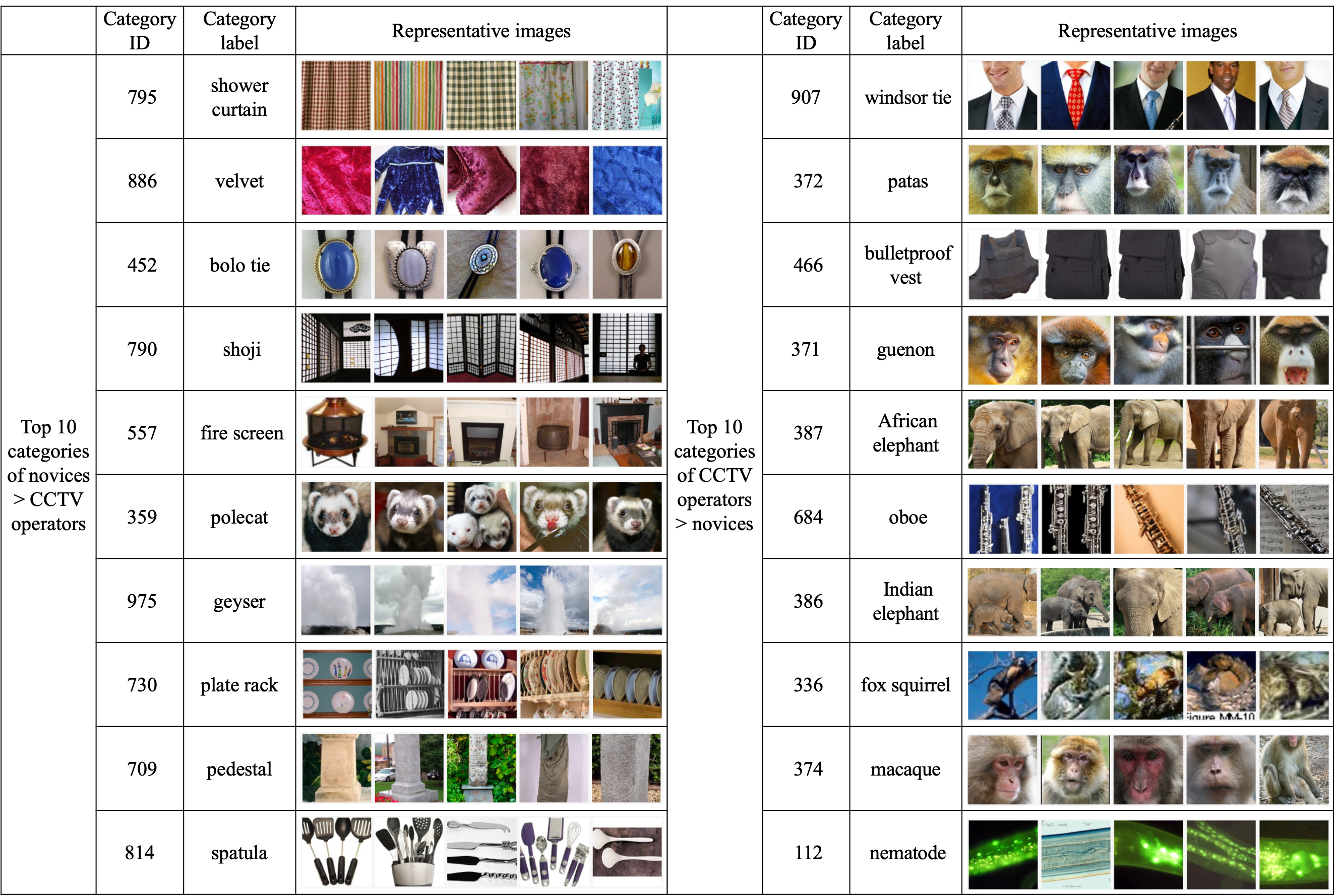


***Figure S5****: Top 10 AlexNet output categories and representative images showing the greatest group contrast between novices and CCTV operators. The half panel on the left demonstrates the top 10 object categories showing higher probability in gaze-centered stimuli from novices than CCTV operators. The half panel on the right includes the top object categories showing higher probability in gaze-centered stimuli from CCTV operators than from novices.* *For novices, the top 10 categories that novices showed greater probabilities than CCTV operators included four categories (795, 886, 790, 975) that were related to texture and color, five categories that were related to manmade objects (452, 557, 730, 709, 814), and one category related to animal (359). In contrast, for CCTV operators, among the top 10 categories that operators showed greater probabilities than novices during video observation included two categories related to clothing (907, 466), three categories related to monkey faces (371, 372, 384), four categories related to animal (386, 387, 336, 112), and one category of a music instrument (684).*
